# Examination of *ex-vivo* viability of human adipose tissue slice culture

**DOI:** 10.1371/journal.pone.0233152

**Published:** 2020-05-26

**Authors:** Nikolas Schopow, Sonja Kallendrusch, Siming Gong, Felicitas Rapp, Justus Körfer, Martin Gericke, Nick Spindler, Christoph Josten, Stefan Langer, Ingo Bechmann

**Affiliations:** 1 Institute of Anatomy, University Leipzig, Leipzig, Germany; 2 Department for Orthopedics, Trauma Surgery, and Reconstructive Surgery, University Hospital Leipzig, Leipzig, Germany; 3 GSI Helmholtzzentrum für Schwerionenforschung GmbH, Darmstadt, Germany; 4 University Cancer Center Leipzig (UCCL), University Hospital Leipzig, Leipzig, Germany; Weill Cornell Medical College in Qatar, QATAR

## Abstract

Obesity is associated with significantly higher mortality rates, and excess adipose tissue is involved in respective pathologies. Here we established a human adipose tissue slice cultures (HATSC) model *ex vivo*. HATSC match the *in vivo* cell composition of human adipose tissue with, among others, mature adipocytes, mesenchymal stem cells as well as stroma tissue and immune cells. This is a new method, optimized for live imaging, to study adipose tissue and cell-based mechanisms of obesity in particular. HATSC survival was tested by means of conventional and immunofluorescence histological techniques, functional analyses and live imaging. Surgery-derived tissue was cut with a tissue chopper in 500 μm sections and transferred onto membranes building an air-liquid interface. HATSC were cultured in six-well plates filled with Dulbecco’s Modified Eagle’s Medium (DMEM), insulin, transferrin, and selenium, both with and without serum. After 0, 1, 7 and 14 days in vitro, slices were fixated and analyzed by morphology and Perilipin A for tissue viability. Immunofluorescent staining against IBA1, CD68 and Ki67 was performed to determine macrophage survival and proliferation. These experiments showed preservation of adipose tissue as well as survival and proliferation of monocytes and stroma tissue for at least 14 days *in vitro* even in the absence of serum. The physiological capabilities of adipocytes were functionally tested by insulin stimulation and measurement of Phospho-Akt on day 7 and 14 *in vitro*. Viability was further confirmed by live imaging using Calcein-AM (viable cells) and propidium iodide (apoptosis/necrosis). In conclusion, HATSC have been successfully established by preserving the monovacuolar form of adipocytes and surrounding macrophages and connective tissue. This model allows further analysis of mature human adipose tissue biology *ex vivo*.

## Introduction

The prevalence of overweight and obesity, commonly measured by a high body mass index (BMI), is notably increasing worldwide [1]. Meanwhile, current research has identified a high BMI as an underlying risk factor for many severe chronic diseases [2]. Furthermore, epidemiologic studies provide clear evidence that, relative to normal weight, obesity is associated with significantly higher all-cause mortality [3]. In the USA alone, a morbid BMI is responsible for approximately 18% of all deaths in the age group of 40 to 85 year-olds [4]. Lastly, prolonged caloric excess leads to adipose tissue remodeling and thereby to chronic inflammation including type 2 diabetes [5,6], cardiovascular diseases [7,8], cancer [9,10], osteoporosis [11], chronic kidney disease [12] as well as depression [13], which altogether constitute the leading causes of disability and death in developed countries.

Although, in general, there are three different types of adipocytes differentiated (white, brown, beige) [2], white adipose tissue seems to occur most frequently [14]. Adipose tissue is an active metabolic and inflammatory organ [15,16]. It can emit adipokines as well as anti- and pro-inflammatory cytokines, e.g. tumor necrosis factor (TNF) and variable interleukins [17,18]. Connected to the chronic inflammation, leukocytosis in adipose tissue has been observed, including macrophages [19,20], B cells [21], T cells [22], neutrophils [23], eosinophils [24], and mast cells [25].

Despite the apparent key role of adipose tissue in severe diseases, only few methodological approaches are available to study its biology *ex vivo*. Historically, fat cells could be isolated and first cell lines, mainly of rodent origin, were obtained [26–28]. However, these cell lines created differentiated adipocytes with multiple lipid droplets. Most frequently used are mice embryo-originated 3T3-L1, 3T3-F442A and C3H10T1/2 cells and DFAT-GFP cells derived from mature adipocytes of GFP transgenic mice [26,29–32].

A monovacuolar state was not reached until the invention of the ceiling cultures by Sugihara et al. in 1986. With this method adipocytes were incubated floating on top of completely filled culture flasks [33]. However, floating adipocytes cultures rapidly dedifferentiate into fibroblast-like cells [34–36]. Three-dimensional culture of isolated mature adipocytes and ceiling culture of adipose tissue fragments were also established by Sugihara [37,38]. These methods allow to investigate proliferation, differentiation and adipocyte functions of mature adipocytes and preadipocytes *in vitro* [39]. A combination of adipose tissue fragments derived from rats in three-dimensional collagen gel was described by Sonoda et al. 2008. They make it possible to observe regenerating preadipocytes and mesenchymal stem cells [40,41]. The first successful experiments on the cultivation of human adipose tissue explants were published by Smith in 1971, and recently Harms et al. published an advancement of the ceiling culture method with mature human adipocytes [42,43]. In recent years, first tissue engineering methods for human adipose tissue have been developed [44,45].

However, interspecies discrepancies impede possible translations of research findings. Amongst numerous obstacles, the significant negative correlations in gene regulation between mice and humans in caloric restriction make direct comparison prone to errors [46]. Further differences occur between sex and life stages. It could, for example, be found that the femoral adipose tissues of premenopausal females appear to have a greater capacity for adipose expansion via hyperplasia, hypertrophy, and insulin sensitivity compared to age-matched postmenopausal females [47].

In addition, the place of origin affects cell composition and the extracellular matrix [48]. Especially the non-cellular structure of the extracellular matrix seems to have a major impact on adipocyte metabolisms and is thus remodeled in diseases (e.g. in diabetes) pointing out the complexity of adipose tissue and the shortcomings of frequently used cell culture models [49,50].

Based on the previously established slice cultures of human tumor tissues, this study aims at investigating whether human adipose tissue can be kept in a human slice culture system model [51–54].

## Materials and methods

### Tissue samples

This study has been approved by the Ethical Committee at the Medical Faculty, Leipzig University (#290-13-07102013). All patients declared their informed consent in written form. Subcutaneous AT was obtained from the Department of Orthopedics, Trauma Surgery, and Plastic Surgery (University Hospital Leipzig, Germany). ATs were derived from abdomen, dorsum, mamma, pelvis and thigh (Table 1). The samples were transported in sterile Hanks’ Balanced Salt Solution (HBSS, Gibco, Life Technologies, Carlsbad, USA) or DMEM (Gibco) and were processed within one to six hours after dissection.

**Table 1:** Adipose tissue samples.

Sample	Origin	Indication of surgery	Age [years]	Sex	BMI [kg/m^2^]	Secondary diagnoses	Medium	Max. Period [days]
#001	Abdomen	Postbariatric	55	male	29	HT, HU, NIDDM, O	I, II, III	14
#002	Mamma	Gynecomastia	21	male	32	-	I, II, III	14
#003	Abdomen	Postbariatric	70	female	34	CAD, NIDDM	I, II, III	14
#004	Abdomen	Postbariatric	59	male	40	CAD, HT, HU, PHT, T2D	I, III	14
#005	Mamma	Gynecomastia	17	male	27	PHT	I, III	14
#006	Dorsum	Postbariatric	34	male	58	HT	I, III	14
#007	Thigh	Postbariatric	62	female	31	HT	I, III	21
#008	Dorsum	Postbariatric	42	female	30	DL, HT, NIDDM	I, III	14
#009	Mamma	Macromastia	36	female	24	-	I, III	14
#010	Dorsum	Postbariatric	25	male	36	-	I, III	14
#011	Abdomen	VRAM flap	57	male	25	CKD, PAD	I, III	14
#012	Abdomen	Postbariatric	52	female	31	PHT	I, III	14
#013	Abdomen	Postbariatric	52	male	29	CAD, DL	I, III, IV, V, VI	14
#014	Abdomen	Postpartum	32	female	22	-	I, III, IV, V, VI	14
#015	Abdomen	Postbariatric	41	female	29	-	I, III, IV, V, VI	14
#016	Abdomen	Postbariatric	47	female	30	NIDDM, PHT	I, III, IV	14
#017	Thigh	Postbariatric	46	male	26	-	I, III	14
#018	Abdomen	Postbariatric	33	male	35	HT	I, III	14
#019	Pelvis	Sarcoma	37	male	23	-	I, III	14
#020	Dorsum	Sarcoma	77	male	28	COPD, HT, HU, PHT	I, III, IV	14
#021	Thigh	Sarcoma	47	female	41	MS, PHT	I, III, IV	14
#022	Pelvis	Exostosis	23	male	22	-	I, III, IV	14

Coronary artery disease (CAD), chronic kidney disease (CKD), chronic obstructive pulmonary disease (COPD), dyslipidemia (DL), hypertension (HT), hyperuricemia (HU), multiple sclerosis (MS), non-insulin-dependent diabetes mellitus (NIDDM), osteoporosis (O), peripheral artery disease (PAD), primary hypothyroidism (PHT), insulin-dependent type 2 diabetes mellitus (T2D), vertical rectus abdominis myocutaneous (VRAM).

### Preparation of slice culture

**Fig 1:**
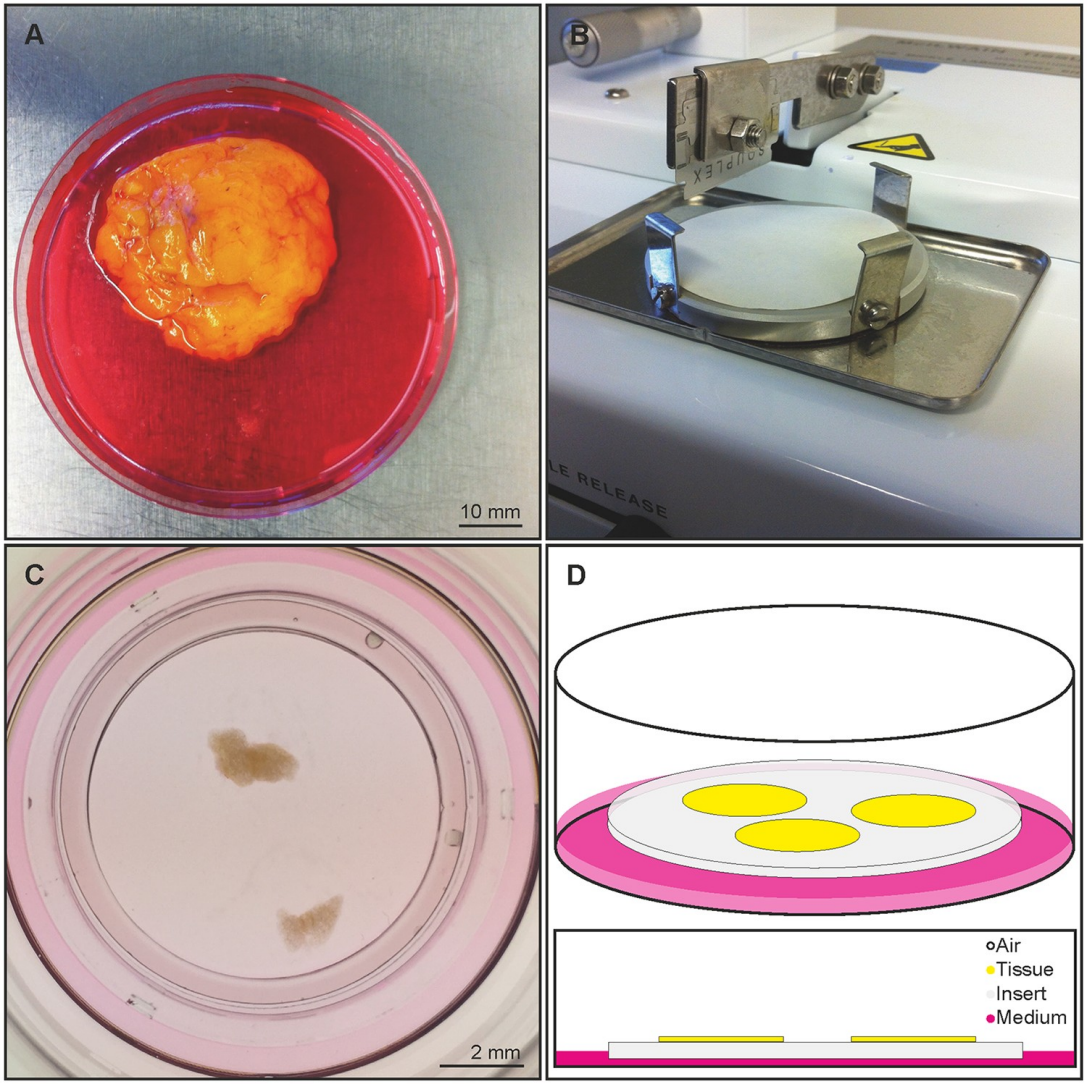
Experimental setup. Tissue samples were derived from orthopedic, trauma and plastic surgeries. A—Tissue was transported in sterile culture medium into the lab. B—Samples were cut into 500 μm thick slices by a tissue chopper. C & D—The slices were incubated on top of filter membrane inserts on a liquid-air-interface in a humidified incubator. On defined points of time specimens were live imaged or fixed.

AT samples were dissected into 5 x 5 x 10 mm pieces and cut by a tissue chopper (Mc Ilwain, Redding, USA) in 350, 500, and 750 μm thick slices. Subsequently, slices were transferred onto cell culture inserts with a pore size of 0,4 μm (Millipore, Merck, Darmstadt, Germany) placed in six-well plates (Corning, New York, USA), and cultivated on a liquid-air-interface in a humidified incubator at 35 °C and 5% CO_2_ (Fig 1). Each well contained 1 ml culture medium under the membrane inserts supplying the tissue via diffusion. The basic culture media consisted of DMEM, insulin-transferrin-selenium mixture (1:100, ITS, Sigma Aldrich, Saint Louis, USA), and Penicillin/Streptomycin (1:100, PenStrep, Gibco). Fetal bovine serum (1:10, FBS, Gibco) or human tumor necrosis factor alpha (50 ng/ml, TNFα, Pepro Tech, Rocky Hill, USA) were added (Table 2). The culture media were changed first after 24 hours, subsequently every 48 hours. Pictures were taken with an Olympus SZ61 Stereomicroscope (Olympus, Tokyo, Japan). Slices, which were fixed at the preparation day, were labeled as day 0. Each culture time point was compared with day 0 and is represented by an individual slice.

**Table 2:** Culture media.

Condition	Basic Medium	Serum	Other contents	Addition
I	DMEM	0% FBS	ITS, PenStrep	
II	DMEM	5% FBS	ITS, PenStrep	
III	DMEM	10% FBS	ITS, PenStrep	
IV	DMEM	0% FBS	ITS, PenStrep	TNFα
V	DMEM	0% FBS	ITS, PenStrep	K^+^
VI	DPBS	0% FBS	ITS, PenStrep	

Dulbecco’s Modified Eagle’s Medium (DMEM), Dulbecco’s phosphate-buffered saline (DPBS), fetal bovine serum (FBS), insulin-transferrin-selenium mixture (ITS), Penicillin/Streptomycin (PenStrep), human tumor necrosis factor alpha (TNFα), potassium ion (K^+^).

### Live imaging

15 minutes prior to imaging the media were removed and replaced by basic culture media containing fluorescent dyes: Hoechst 33342 (Nuclei, 1:1000, Sigma Aldrich), propidium iodide (apoptosis/necrosis, 1:1000, PI, Calbiochem, Darmstadt, Germany) and Calcein-AM (unspecific metabolism 1:200, Life Technologies). Pictures for 3D reconstruction or videos were taken with an Olympus IX81 confocal microscope (FV1000, Olympus) equipped with a humidified incubator and a motorized stage. During the imaging procedure, inside temperature was adjusted to 35 °C, 5% CO_2_, and 60% humidity.

### Tissue analysis

At 0, 1, 7 or 8, and 14 days *in vitro* (DIV), slices were fixed over-night in 4% paraformaldehyde (PFA) prior to paraffin embedding. Paraffin sections (10 μm) were cut, dewaxed in xylene, dehydrated in decreasing alcohol series, and stained with hematoxylin/eosin (H/E) for conventional histology. Pictures were taken using an optical microscope, Axioplan 2 (Carl Zeiss, Oberkochen, Germany). In order to establish immunofluorescence, sections were pretreated with citrate buffer (pH 6) in a microwave for 10 minutes and antibodies were incubated over-night at 4 °C in 1,5% Triton/PBS with 0,5% bovine serum albumin (BSA, Sigma Aldrich) and 10% normal goat serum or normal donkey serum (NGS or NDS, Jackson Immuno Research, West Grove, USA). To observe proliferation, antibodies against Ki67 (1:400, rabbit, DCS, Hamburg, Germany) were used. Apoptosis was detected by staining for activated Caspase-3 (1:300, rabbit, Cell Signaling, Cambridge, United Kingdom). Macrophages were labeled using Anti-IBA1 (1:500, rabbit, Wako, Osaka, Japan; 1:500, guinea pig, Synaptic Systems, Göttingen, Germany) or Anti-CD68 (1:100, mouse, DAKO, Agilent, Santa Clara, United States). Viability of adipocytes was visualized using Perilipin A (1:500, rabbit; 1:250, goat, both Abcam, Cambridge, United Kingdom). The sections were washed and incubated at room temperature with secondary antibodies for one hour (1:500, goat-anti-rabbit Alexa 488/568; 1:500, goat-anti-guinea pig Alexa 488; 1:250, donkey-anti-rabbit Alexa 488; 1:250, donkey anti-goat Alexa 568, all Life Technologies). Nuclei were counterstained using Hoechst 33342 (1:10.000, Sigma-Aldrich). Pictures were taken using a fluorescence microscope BX40 (Olympus) or a LSM 710 (Carl Zeiss). In order to compare the cell size between conditions, the cross-section area of 20–60 adipocytes of each H/E stained section per condition of four experiments (#009, #010, #011 and #021) was measured manually using ImageJ (Version 1.8.0).

### Western blot

48 hours prior to the experiment, adipocyte tissue cultures were serum starved overnight by changing medium to serum and insulin free medium. To determine Akt phosphorylation, adipocytes were stimulated with insulin (10 nM), diluted in pre-warmed serum free medium for 15 min. Separation of membranes and cytosol was performed by a protocol modified from Nishiumi and Ashida [55]. Briefly, adipocyte cultures were collected in buffer A (50 mmol/l Tris, 0.5 mmol/l dithiothreitol, adjusted to pH 8.0 and 1% phenylmethylsulfonyl fluoride (PMSF), 10 mM sodium orthovanadate and 1% Sigma protease inhibitor cocktail freshly added) and stored at -80 °C until further analysis. Western blot analysis was performed as described earlier [56]. Blots were incubated with Phospho-Akt (1:1000, Cell Signaling) at 4 °C overnight. Immunoreactions were detected with the appropriate peroxidase-conjugated anti-rabbit IgG secondary antibody (1:5000 for phospho-specific antibodies; Vector Laboratories, Peterborough, UK) at room temperature for 2 h. Peroxidase activity was visualized with an enhanced chemiluminescence kit (Amersham, Pharmacia, Freiburg, Germany). In addition, blots were stripped and incubated with pan-Akt antibody (1:3000, Cell Signaling) followed by a secondary antibody (1:10000, anti-rabbit IgG, Vector Laboratories). In addition, blots were stripped and incubated with anti-glyceraldehyde-3-phosphate dehydrogenase (GAPDH) monoclonal antibody (diluted 1:100000, Research Diagnostics, Flanders, Netherlands) followed by the anti-mouse IgG secondary antibody (1:10000, Vector Laboratories). GAPDH antibodies were used as a loading control. Semiquantitative evaluation of arbitrary unit was performed with the ImageJ plugin for western blot analysis.

### Statistical analysis

One-way-ANOVA with Bonferroni correction was performed using GraphPad Prism 6 (GraphPad Software, Inc., La Jolla, USA). P <0.05 was considered significant.

## Results

Adipose tissues were derived from nine abdominal, four dorsal, three breast, three pelvic, and three limb surgeries (Table 1) and were kept in culture for up to 14 days. The tissue donors were 43,9 years (SD ±15,9) old on average, had a BMI of 31,0 kg/m^2^ (SD ±8,0) on average, and 40,9% were female. In 14 cases, the patients had secondary diagnoses (Table 1). Tissue were transferred from surgery and cut on a tissue chopper between 1 and 6 hours after removal (Fig 1). Pioneering experiments investigating the optimal thickness for cultivation showed that 500 μm were ideal for tissue preparation and handling. 350 μm thick slices often collapsed during the preparation process while 750 μm slices proved difficult to embed into paraffin for further histological analysis. 500 μm thick slices maintained approximately five to seven cell layers and all layers were well preserved during cultivation.

**Fig 2:**
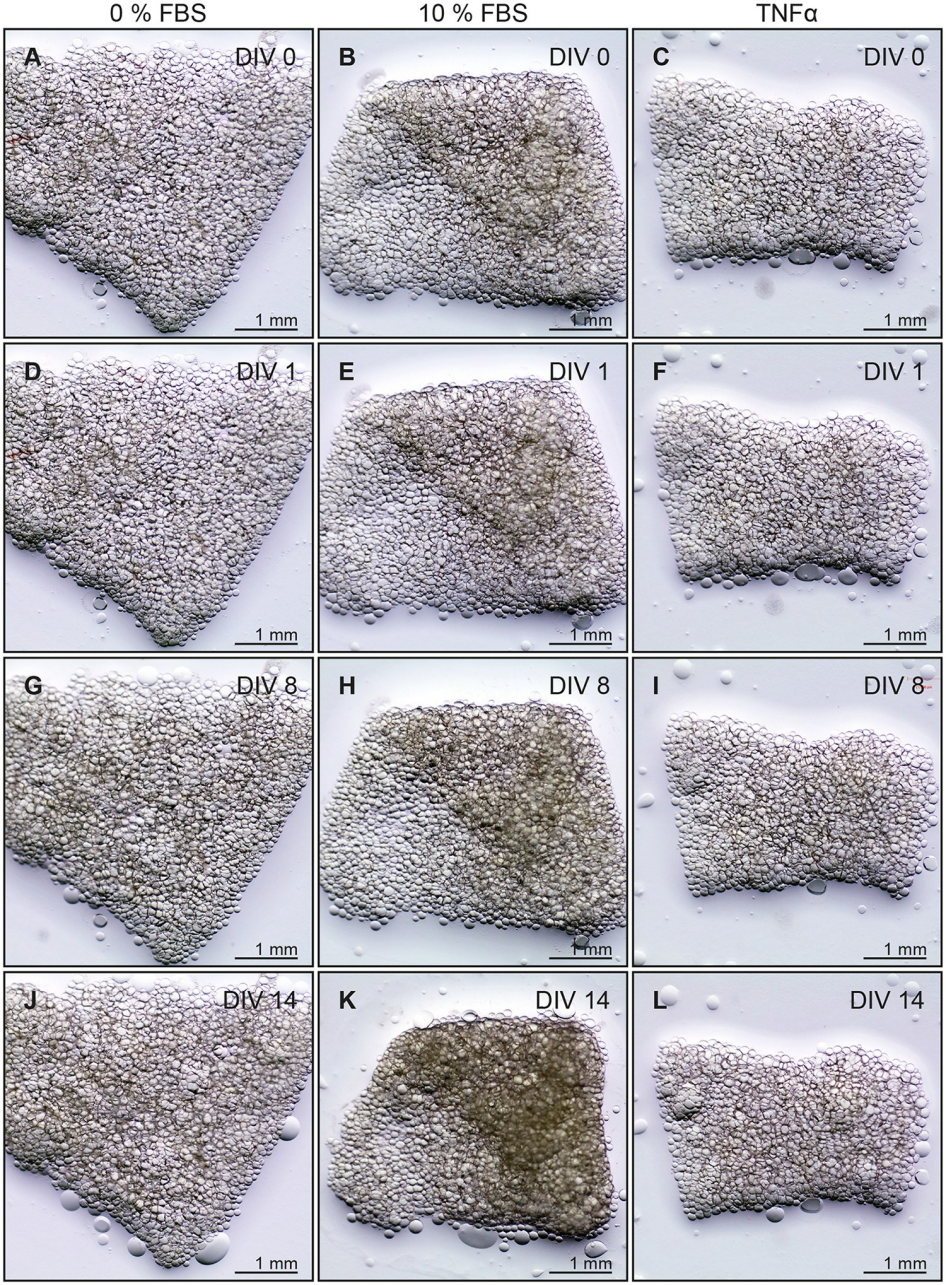
Macroscopic development of adipose tissue under different culture conditions. On defined points of time in each case the same slice was photographed using a Zeiss Stereomicroscope. A to C—0^th^ DIV. D to F—1^st^ DIV. G to I—8^th^ DIV. J to L—14^th^ DIV. Left column—0% FBS. Middle column—10% FBS. Right column—TNFα.

Tissue integrity was macroscopically well preserved up to 14 DIV and adipocyte appearance did not change (Fig 2). Tissue slices cultivated with 10% FBS (Table 2, III) showed minor slice shrinkage (Fig 2B, 2E, 2H and 2K). To induce distinct tissue damage, TNFα was supplemented to serum-free media provoking cell death (Figs 2C, 2F, 2I, 2L and 4).

**Fig 3:**
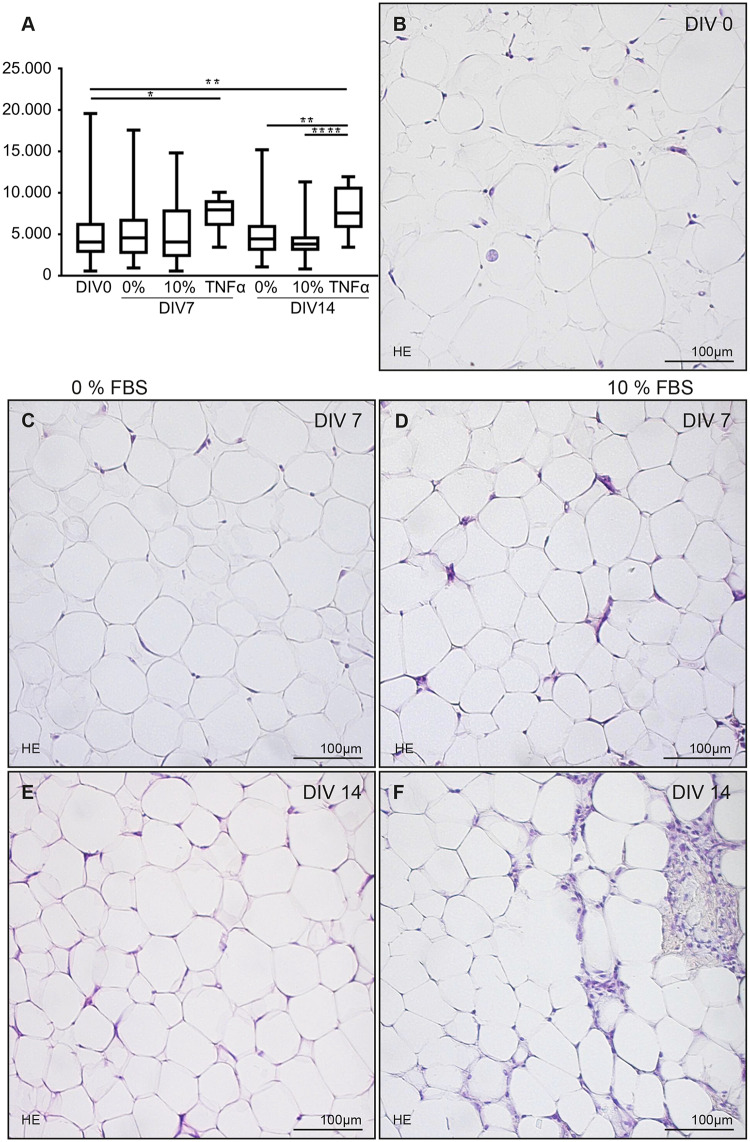
Tissue analysis. A—No measurable difference in the cross-section area of adipocytes in medium with and without 10% FBS, but TNFα supplementation increased the cross-section area of adipocytes (y-axis in μm^2^). Morphological analysis was performed via H/E staining and showed well-sustained AT. B—0^th^ DIV. C & D—7^th^ DIV. E & F—14^th^ DIV. Left column—0% FBS. Right column—10% FBS.

Histological analysis of H/E staining’s demonstrated the well-maintained characteristics of AT (Fig 3). The cross-section area of adipocytes increased under TNFα supplementation, while no measurable difference was observed between the two different culture media between 0 and 14 DIV (Fig 3). Slices cultivated without serum supplementation (I, Table 2) maintained their cellular composition up to 14 DIV. In medium supplemented with serum, stroma tissue appeared to expand, but no obvious discrepancy could be observed between conditions with a dose of 5% FBS (II, Table 2) as compared to 10% FBS (III, Table 2) (S1 Fig). Taking into consideration the high serum doses used in previous experiments (e.g. 15% FBS [57]), 10% FBS was used for our further experiments.

**Fig 4:**
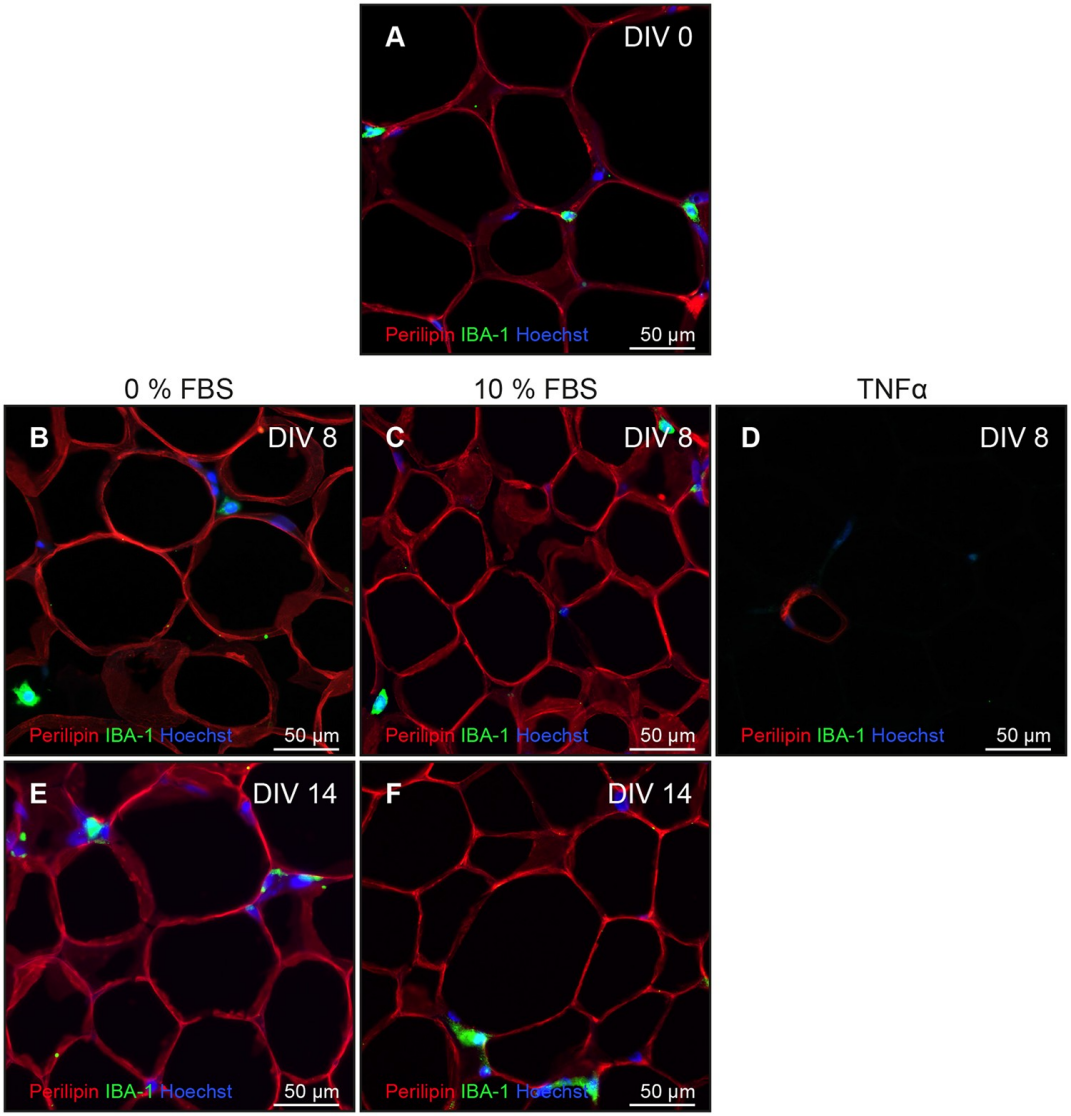
Viability of adipose cells. Viability was determined via immunofluorescence and insulin stimulation. Antibodies against Perilipin (red) and monocytes were shown with IBA1 (green). Cell nuclei were counterstained (Hoechst 33342, blue). A—0^th^ DIV. B to D—8^th^ DIV. E & F—14^th^ DIV. Left column—0 % FBS. Middle column—10 % FBS. Right column—human TNFα.

**Fig 5:**
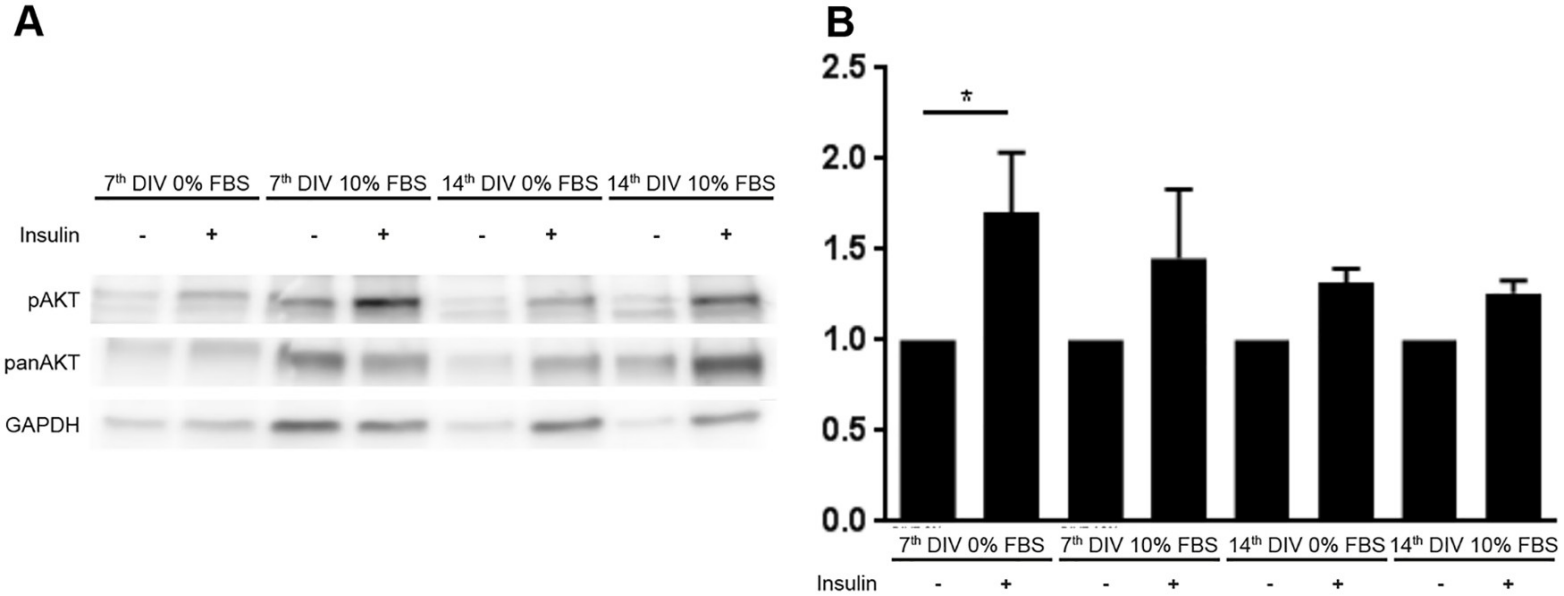
Protein expression in HATSC. Tissue cultured with and without 10% FBS on 7^th^ and 14^th^ DIV. A—Representative western blot for Phosphor-Akt, pan-Akt and GAPDH. B–Quantitative analysis (n = 4) of the protein expression levels normalized to pan-Akt. From four individual donors, three slices were used for each condition and time point for the analysis.

**Fig 6:**
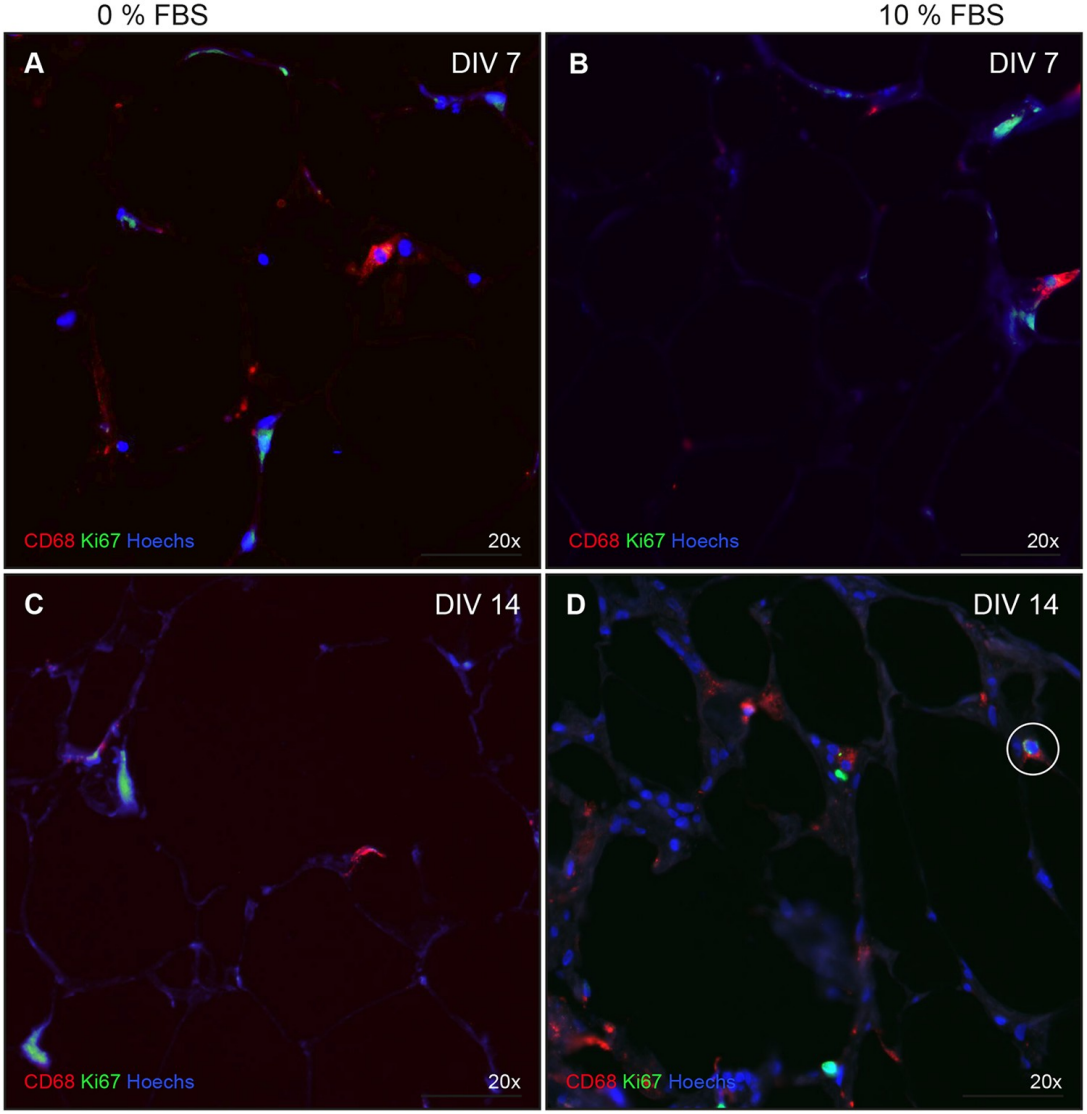
Proliferation and inflammation. Proliferation and inflammation in abdominal tissue were determined via immunofluorescence with antibodies against Ki67 (green) and CD68 (red). Cell nuclei were counterstained (Hoechst 33342, blue). A & B—7^th^ DIV. C & D—14^th^ DIV. Left column—0% FBS. Right column—10% FBS. Circle triple positive cell -> Proliferating macrophage.

Viability of adipose cells was determined via immunofluorescence with antibodies against Perilipin A (Fig 4). No obvious differences between the endpoints of the standard conditions I and III (Table 2) could be observed. Homogeneous expression of the lipid droplet surface protein in both conditions proved the survival of adipocytes in slice cultures for 14 days. As a positive control for cell death, TNFα was added to the medium (IV, Table 2; Fig 4D). Thus, positive controls confirmed the predictive validity of Perilipin A. To further investigate function of adipocytes the phosphorylation of Akt, a key step in insulin signaling, was investigated after 15 min of insulin stimulation. After 7 and 14 DIV the phosphorylation of Akt remains low in the control condition, whereas the insulin condition shows an enhanced expression of phosphorylated Akt (Fig 5A & 5B). Using antibodies against IBA1 and CD68 revealed viability of macrophages. Some CD68-positive macrophages were co-localized with Ki67, proving proliferation processes on 14^th^ DIV (Fig 6, Circle).

**Fig 7:**
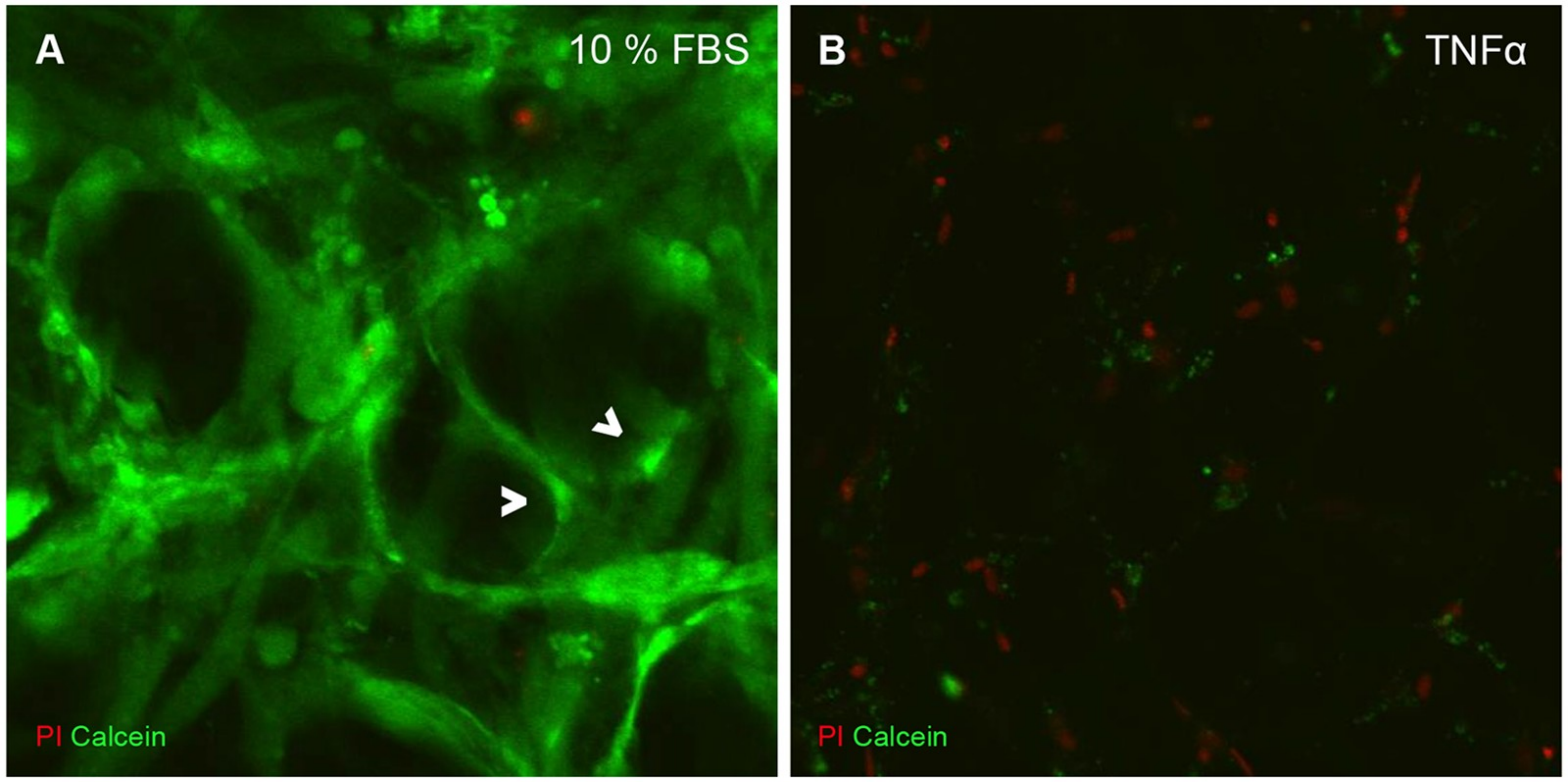
Live imaging of slice cultures on DIV 14. Viability and death of cells were determined via Calcein-AM (cell metabolism, green) and propidium iodide (apoptosis/necrosis, red). A– 14^th^ DIV, 10% FBS. B– 14^th^ DIV, TNFα. Calcein positive cytoplasm of adipocytes exemplarily marked with arrows.

In live imaging analyses medium with Calcein-AM was added 15 minutes prior to taking pictures. The non-fluorescent Calcein-AM diffused through cell membranes, intracellular esterases hydrolyzed the acetoxymethylester-group, and the fluorescent Calcein accumulated in cell plasma [58]. This activating process could be shown in vital adipocytes and stroma cells on day 7 and 14 *in vitro* (Fig 7, S1 Video). However, exposing the tissue to laser light provoked a positive control cellular death of both adipocytes and stroma cells after prolonged exposure (S2 Fig, S2 Video).

## Discussion and conclusion

Organotypic slice cultures of human adipose tissue maintained their morphological characteristics and their metabolism for up to 14 days in culture. It can thereby be concluded that HATSC provides a platform to investigate human adipose tissue in a controlled *ex vivo* setting with little ethical constraints.

Morphological analysis of H/E staining’s cannot discriminate viable adipocytes from dead adipocytes; The distinction between living and dead adipocytes (lipid droplets with or without cell nuclei) cannot be made on the basis of cell nuclear morphology. In histologic standard sections (approx. 10 μm) of normal-sized adipocytes (approx. 50–150 μm) their small nucleus is not necessarily part of the histological section. Therefore, the state of a given adipocyte cannot always be judged in the basis of its morphology (e.g. euchromatic, pyknotic, fragmented). Studies in the literature on changes in the cell volume of adipocytes as a distinguishing feature of vitality or apoptosis/necrosis of adipocytes do not exist. Jo et al. did not find an alteration in cell volume during apoptosis, using mathematical models [59]. They could show that under prolonged weight-loss conditions large adipocytes shrink, but at the same time the smaller adipocytes die first [60]. A faster cell death of the small adipocytes could lead to an increase in the average cell volume, even if the large adipocytes themselves shrink. In a human study, Verboven et al. could show that people with obesity, i.e. those with an increased inflammation in fatty tissue, have more large and very large adipocytes, although they have an increased basal lipolysis [61]. They attribute this to a decrease in the number of small adipocytes. Both processes, early cell death of small adipocytes and the slow shrinkage of large adipocytes could explain the total increase in adipocyte surface area of the TNFα condition that was observed in the present study (see Fig 3A).

The survival of adipocytes was demonstrated by immunofluorescence staining and the viability of adipose tissue by live imaging and functional experiments. Perilipins are lipid droplet-associated proteins and their phosphorylation is essential in lipolysis. Perilipin A is a known marker for viability of adipocytes and has been used in immunological research [62–66]. Using our own experiments, we were able to confirm the sensitivity of Perilipin A as a viability marker of adipocytes (see Fig 4A and 4D).

Pathological remodeling of adipose tissue includes hypertrophy, accumulation of immune cells such as macrophages, decreased capillary density, and fibroblast activation [67]. There is some evidence that adipose tissue is able to control local regulation and proliferation of macrophages independently of the influx of blood precursors, but no evidence currently exists supporting the role of local myelopoiesis in adipose tissue [68–72]. The preservation of macrophages in HATSC was displayed with immunofluorescence staining with IBA1 and CD68. Both antibodies were selected for their wide distribution in the study of macrophages in adipose tissue [73,74]. Even the local proliferation in the absence of blood could be demonstrated on DIV 14 in human adipose tissue, where CD68-positive macrophages were co-localized with Ki67 (Fig 6).

Live-imaging was used to further study the formation of crown-like structures consisting of proliferating macrophages around dying adipocytes in murine adipose tissue [75,76]. The results show that such experiments could also be performed in human tissues. In a rodent study by Weisberg et al. TNFα was shown to be distributed by macrophages and not by adipocytes as part of the stroma-vascular fraction during inflammation and diabetes [19,77]. Contrary to this, human adipocytes have the potential to secrete TNFα, thus signaling to immune cells [78–80]. Such potential species differences can now be worked out. Moreover, studying human adipose tissues from individuals with different BMI with and without type II diabetes might help to better understand how inflammation and oxidative stress drives insulin resistance, arteriosclerosis, angiogenesis, as well as cancer [81–84]. This can be concluded since the open access of the system allows for studies of the accumulation of (secreted) molecules in the medium. The large number of standardized samples which can be prepared from small probes further enables the investigation of therapeutics, targeting essential biochemical pathways, drug accumulation, and modern pharmaceutics for gene therapy [85].

In conclusion, slice cultures derived from human adipose tissue have been successfully established whereas the unique monovacuolar shape of the adipocytes as well as the complex organization of the tissue could be maintained. Hence, this method serves as a confirmation of the current findings about rodent adipose tissue while it allows to further dissect its biology in the human system.

## Supporting information

S1 Fig Analysis of different culture media. Analysis was performed via H/E staining. Top row—0% FBS, middle row—5% FBS and bottom row—10% FBS. A, C, E– 1^st^ DIV. B, D, F—8^th^ DIV. The adipose tissue slice cultures maintain most of their morphologic properties, but high serum concentrations increased the fibrocyte fraction.
(TIF)

S2 Fig Live imaging of slice cultures on DIV 0. Viability and death of cells were determined, directly after preparation of slice cultures in basic media without serum, via Calcein-AM (cell metabolism, green) and propidium iodide (apoptosis/necrosis, red), e.g. arrows. A to C—60 min; D to F—180 min; H to J—300 min after laser exposure.
(TIF)

S1 Video Live imaging of slice cultures on 7^th^ DIV (0% FBS). Viability and death of cells were determined via Calcein-AM (cell metabolism, green) and propidium iodide (apoptosis/necrosis, red).
(AVI)

S2 Video Live imaging of slice cultures on 0^th^ DIV. Viability and death of cells were determined via Calcein-AM (cell metabolism, green) and propidium iodide (apoptosis/necrosis, red), directly after preparation of slice cultures in basic media without serum.
(AVI)
