# Supplementary figures and images for: Examination of ex-vivo viability of human adipose tissue slice culture
Source: PLoS One. 2020 May 26;15(5):e0233152. doi: 10.1371/journal.pone.0233152 (PMC7250419; doi:10.1371/journal.pone.0233152)

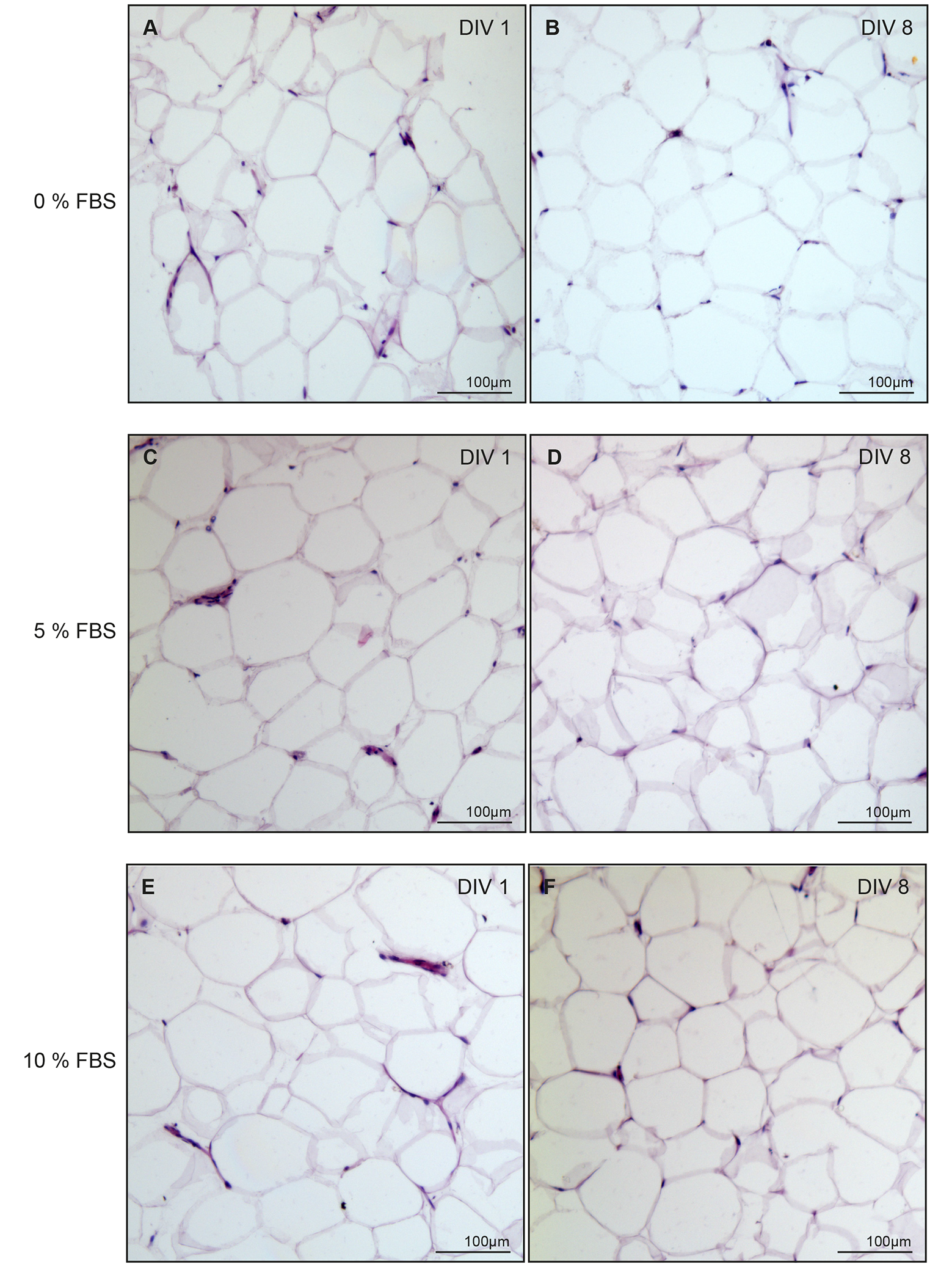

Supplement: S1 Fig — Analysis was performed via H/E staining. Top row—0% FBS, middle row—5% FBS and bottom row—10% FBS. A, C, E– 1st DIV. B, D, F—8th DIV. The adipose tissue slice cultures maintain most of their morphologic properties, but high serum concentrations increased the fibrocyte fraction. (TIF) [file pone.0233152.s001.tif]

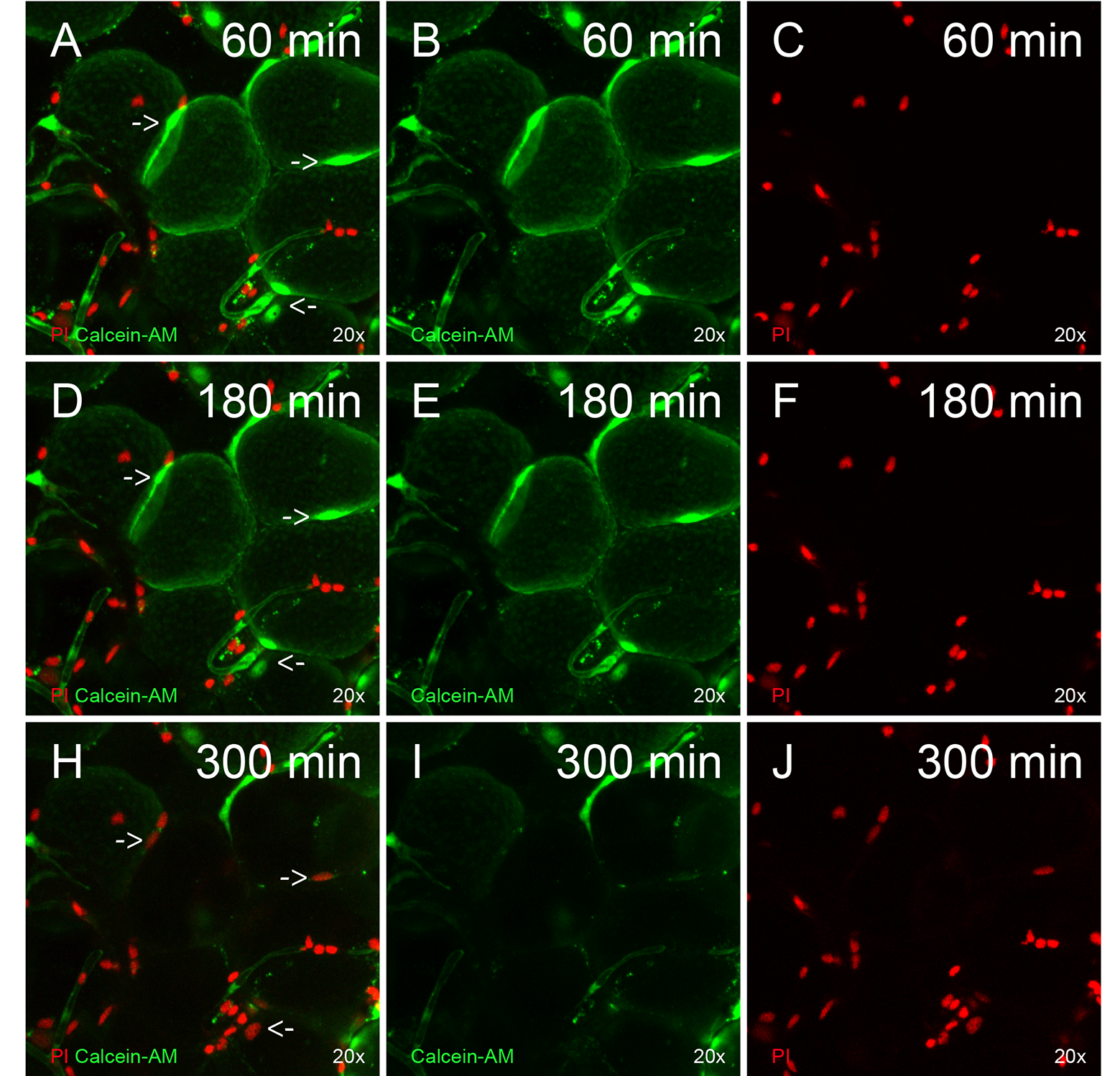

Supplement: S2 Fig — Viability and death of cells were determined, directly after preparation of slice cultures in basic media without serum, via Calcein-AM (cell metabolism, green) and propidium iodide (apoptosis/necrosis, red), e.g. arrows. A to C—60 min; D to F—180 min; H to J—300 min after laser exposure. (TIF) [file pone.0233152.s002.tif]
